# Development of an Ecological Momentary Assessment Mobile App for a Low-Literacy, Mexican American Population to Collect Disordered Eating Behaviors

**DOI:** 10.2196/publichealth.5511

**Published:** 2016-07-14

**Authors:** Kay Connelly, Karen F Stein, Beenish Chaudry, Nicole Trabold

**Affiliations:** ^1^ School of Informatics and Computing Indiana University Bloomington, IN United States; ^2^ School of Nursing University of Rochester Rochester, NY United States; ^3^ Notre Dame South Bend, IN United States; ^4^ Department of Psychiatry Medical Center University of Rochester Rochester, NY United States

**Keywords:** feeding and eating disorders, health literacy, socioeconomic status, human-centered computing, user-computer interface, mobile apps

## Abstract

**Background:**

Ecological momentary assessment (EMA) is a popular method for understanding population health in which participants report their experiences while in naturally occurring contexts in order to increase the reliability and ecological validity of the collected data (as compared to retrospective recall). EMA studies, however, have relied primarily on text-based questionnaires, effectively eliminating low-literacy populations from the samples.

**Objective:**

To provide a case study of design of an EMA mobile app for a low-literacy population. In particular, we present the design process and final design of an EMA mobile app for low literate, Mexican American women to record unhealthy eating and weight control behaviors (UEWCBs).

**Methods:**

An iterative, user-centered design process was employed to develop the mobile app. An existing EMA protocol to measure UEWCBs in college-enrolled Mexican American women was used as the starting point for the application. The app utilizes an icon interface, with optional audio prompts, that is culturally sensitive and usable by a low-literacy population. A total of 41 women participated over the course of 4 phases of the design process, which included 2 interview and task-based phases (n=8, n=11), focus groups (n=15), and a 5-day, in situ deployment (n=7).

**Results:**

Participants’ mental models of UEWCBs differed substantially from prevailing definitions found in the literature, prompting a major reorganization of the app interface. Differences in health literacy and numeracy were better identified with the Newest Vital Sign tool, as compared with the Short Assessment of Health Literacy tool. Participants had difficulty imagining scenarios in the interviews to practice recording a specific UEWCB; instead, usability was best tested in situ. Participants were able to use the EMA mobile app over the course of 5 days to record UEWCBs.

**Conclusions:**

Results suggest that the iterative, user-centered design process was essential for designing the app to be made usable by the target population. Simply taking the protocol designed for a higher-literacy population and replacing words with icons and/or audio would have been unsuccessful with this population.

## Introduction

### Ecological Momentary Assessment

Ecological momentary assessment (EMA) is a measurement approach rapidly gaining popularity in public health research because of its utility in capturing complex processes associated with major health problems and their treatment. To date, more than 250 studies have used EMA to investigate critical public health problems ranging from diabetes, hypertension, asthma, tobacco, alcohol and drug use, obesity, and physical inactivity. EMA refers to the collection of individuals’ behavioral, emotional, cognitive, and biophysical data as they occur in the natural environment, most frequently using an electronic device. EMA is considered a class of measurement approaches that are unique in that they share a set of three properties: (1) the phenomenon of interest is measured repeatedly over time, (2) measurement occurs within the naturally occurring context, and (3) data are collected as the target event occurs or shortly thereafter [1]. Therefore, EMA eliminates problems associated with retrospective recall of a targeted event (eg, how many cigarettes did you smoke in the last month?) and threats to ecological validity. Rather, EMA provides a detailed picture of the individual’s experiences as they naturally occur over time and across situations.

An important advantage of EMA is that both objective and subjective data related to the target event can be measured simultaneously or in close succession. Although some studies collect biomarker data in vivo in conjunction with self-report data, others rely exclusively on self-report data. For example, in several studies of the effects of stress on health outcomes, salivary cortisol was measured several times daily over the course of several days concurrently with self-report measures of affect and stress [2,3]. Other EMA studies focused solely on participant subjective experience with protocols including multiple word-based questionnaires.

Despite the many strengths of EMA, one notable limitation is that data collection often includes complex protocols, and subjective data collection relies on word-based questionnaires that include complex ratings and self-evaluations [4]. As a result, EMA studies often exclude large and particularly vulnerable segments of our population—persons with low health literacy.

Health literacy is defined as “the ability to obtain, process, and understand basic health information and services needed to make appropriate health decisions and follow instructions for treatment.” Health literacy, like general literacy, address three skill sets including the ability to search, comprehend, and use information from continuous prose (brochures, instructions), noncontinuous text in diverse formats (tables, forms), and quantitative tasks. Health literacy is strongly associated with race, level of education, and socioeconomic status (SES) [5,6]. Furthermore, health literacy is affected by cultural and linguistic factors that further complicate comprehension of both printed and verbally presented materials. Estimates suggest approximately 12% of the US population has less than a high school education [7] and more than 36 million adults living in the United States read at or below the third grade level. In terms of health literacy, 49% of persons with less than a high school diploma demonstrated below basic level healthy literacy [5]. Yet EMA studies rarely report participants’ level of education and those studies that do almost universally include samples of highly educated participants. Virtually no studies report literacy or health literacy levels of study participants.

This methodological gap is of great concern for public health researchers as low-literacy populations often experience significant health disparities [8] and attempts to quantify their experiences are limited by the lack of valid and reliable approaches to measurement. This paper presents a case study of how to design an EMA tool for a low-literacy population.

### Contributions

We present a case study of a user-centered, iterative design process with a Western New York rural farming population of Mexican American (MA) women. The goal of the design process was to develop a culturally sensitive, understandable and usable mobile phone app to measure unhealthy eating and weight control behaviors (UEWCBs) in a sample of MA women with low literacy skills. UEWCBs include binge eating, food or calorie restriction, fasting, self-induced vomiting, and use of diet pills, diuretics, and laxatives as a means to lose or control body weight. We focused on UEWCBs in this population for several reasons. First, the prevalence of these behaviors is high in MA women of higher SES, and results of a few studies of MA women living in border states suggest they also are prevalent in low SES MA women [9]. Second, EMA methodology has been used effectively both to identify determinants of UEWCBs and as an approach to treatment in higher SES majority women. Finally, UEWCBs contribute to weight gain and indicators of metabolic syndrome—both of which disproportionately affect MA women. Alcohol and tobacco use are highly correlated with UEWCBs in majority populations [10] and prevalence of use is increasing in populations of MA women [11]. Therefore, items to measure alcohol consumption and tobacco use were included. 

The case study started with an EMA protocol designed to measure the behaviors of interest in college-enrolled MA women [12]. Although the parent study did not address diagnosable level eating disorders, definitions of the UEWCBs were based on the *Diagnostic and Statistical Manual of Mental Disorders, Fourth Edition* (*DSM-IV*) [13]. Problems with a direct adaptation of the EMA and research protocols to a low-literacy population were identified and rectified through 4 phases of the design process meant to (1) identify icons to visually represent the target behaviors that are understandable and culturally relevant to MA women and (2) develop behavior-recording navigation that is intuitive and easy to use.

## Methods

**Figure 1:**
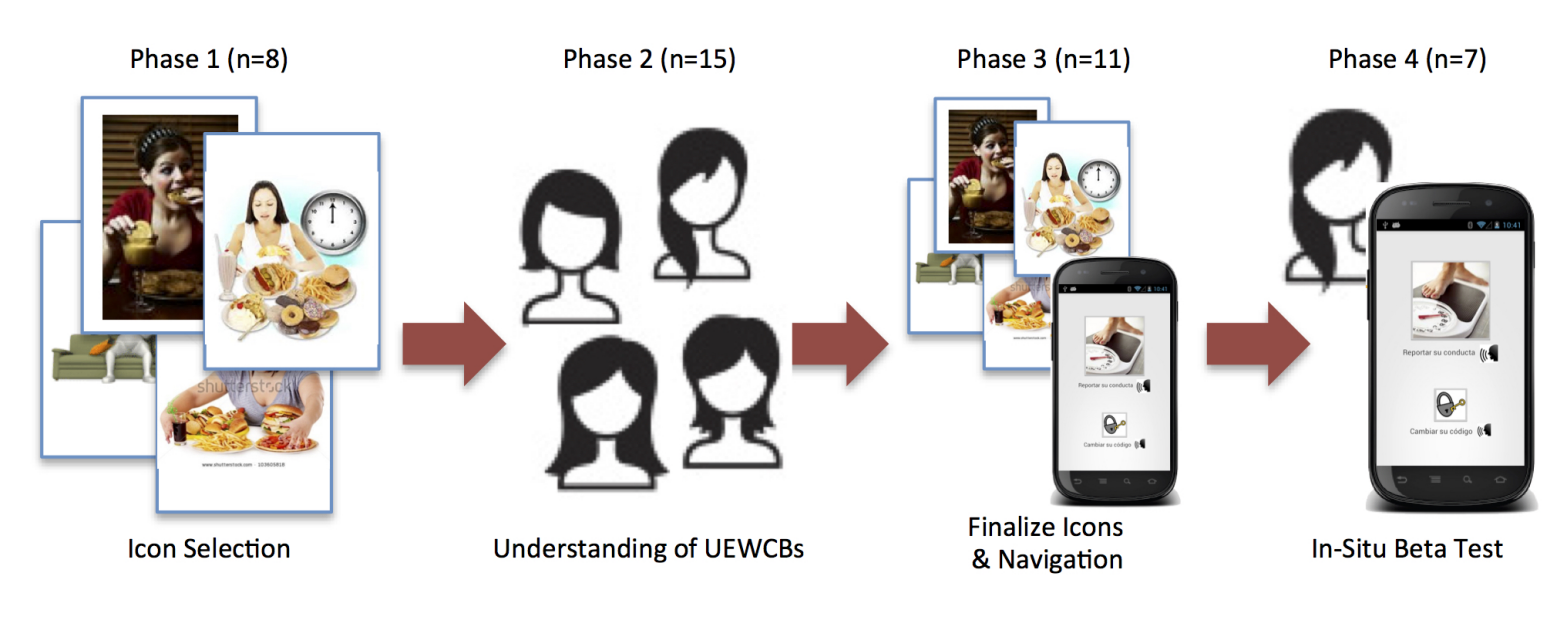
The 4 phases in our final protocol.

The study protocol was approved by the institutional review board (IRB) of the 2 participating universities. Adopting a user-centered, iterative design process meant that we did not know the exact nature of the phases of the study a priori. The final protocol consisted of 4 user-centered phases (Figure 1), including (1) activities to elicit feedback on icons for the interface, (2) a focus group to clarify the population’s understanding of UEWCBs, (3) activities to finalize icons and test the navigation of the app, and (4) an in situ, 5-day beta test with a fully functional app. All data collection activities were conducted in Spanish. A female member of the community who came to the United States as a migrant farm worker and is widely known by the community as a farm worker rights advocate was employed by our project as a community liaison. In addition, a Spanish-English bilingual woman was employed as data collector.

The community liaison and data collector completed protection of human subjects certification and were trained for data collection by the project manager. Training activities were completed before each phase and included the subject recruitment protocol, informed consent process, and data collection activities. Although there were slight variations across the phases, generally the community liaison administered the informed consent and collected demographic data. The data collector administered health literacy measures and all study questionnaires. For the beta test, the data collector oriented the participant to the mobile phone app and behavioral recording. The programmer responsible for developing the mobile phone app participated in the first 2 phases to observe participants’ mobile phone use competency and ability to navigate early versions of the app.

### Translations

All study materials, including the informed consents, interview scripts, and measures, were translated into Spanish by a certified translator with fluency in Mexican Spanish. All translations were reviewed by the community liaison and discrepancies or questions were resolved through discussion with the translator. Responses to open-ended questions were translated into English after the sessions by the data collection team.

### Recruitment

The community liaison assumed responsibility for all recruitment activities, including verbal announcements at local churches, day care centers, farm community social activities, and Mexican Consulate on Wheels programs. In addition, the project had IRB approval allowing the community liaison to recruit via home visits. Regardless of the mode of initial contact, the recruitment process included 2 face-to-face visits at the potential participant’s home. During the first visit, the community liaison described the project to the potential participant and answered questions. The informed consent was read aloud and the participant was asked to spend a few days talking with her family and thinking over her interest in participating. During the second visit, the participant was asked if she would like to participate and, if she did, an appointment was set for the data collection session.

### Data Collection

Except for the focus groups, all data collection sessions were individual, face-to-face interviews conducted at participants’ homes. Both the community liaison and Spanish-speaking data collector conducted all data collection sessions together. Because of issues related to immigration status, names were not recorded on study documents. Informed consent was granted orally with a witness’ signature to document agreement. Participants were monetarily compensated for their time, with the amount varying across study phases. Participants were paid in cash (phases 1-3: US $15, phase 4: US $55) and a witness’ signature documented the payment. Except for one participant who participated in both phase 2 and phase 4, participants were involved in only a single phase of the study.

### Measures

Two measures for heath literacy were used to ensure we were reaching our target population. A custom usability questionnaire was used for the final phase to identify potential problems with the app. Psychometrics of the measures was not computed for this study because of the small sample size.

- Short Assessment of Health Literacy–Spanish and English (SAHL-S&E) is an 18-item measure that tests word reading ability and comprehension in the health context. A score of 0-14 was considered inadequate health literacy. SAHL was used for each study. Correlations between SAHL-S and other measures of health literacy (50-item Short Assessment of Health Literacy for Spanish Adults and the Test of Functional Health Literacy in Adults) and level of education in Spanish-speaking samples were positive and strong. Alpha coefficients for the 18-item English and Spanish versions were .89 and .80, respectively. Results of item response theory indicate that the English and Spanish versions are comparable tests [14].
- Newest Vital Sign (NVS) is a tool to assess health literacy that addresses both reading and numeracy skills [15]. Total scores of 0-1 suggest high likelihood of limited literacy; scores of 2-3 indicate possible limited literacy; scores of 4-6 indicate adequate literacy. Evidence to support construct validity and internal consistency (English: alpha=.76; Spanish: alpha=.69) has been shown [16]. The NVS was used for phases 3 and 4.
- A 43-item usability and context of use questionnaire, modified from [17], was administered at the final interviews for the beta study, which used a working version of the app. Questions focused on factors influencing the recording of behaviors, including overall usability of the app (8) and individual features (icons, 5; reminders, 8; and the camera, 2), learnability and help within the app (5), context of use (11), and how using the app made them feel about their health (4). A total of 31 questions were on a 5-point Likert scale and 12 questions were yes or no answers.

### Analysis

Descriptive statistics were used to analyze data in each phase except phase 2. The sample size for each phase, although appropriate for the specific design activity (eg, Siek et al [18] report a picture card study with n=8), is too small to perform inferential analysis beyond averages and counts. In general, if more than one participant had difficulty with an activity, the associated interface component was discussed by the team and improved upon, or further user feedback was elicited. Focus group audiotapes were transcribed and translated. Conventional content analysis was used to analyze the data [19]. Two investigators (KS and NT) participated in the data coding and analysis. Transcripts were read to achieve initial understanding and followed by a line-by-line analysis to derive codes and themes that reflect concepts and thoughts. After all transcripts were coded, the 2 investigators met to discuss any discrepancies in codes, cluster codes into meaningful categories, and discuss potential relationships. During the final phase, means and standard deviations of the usability questionnaire were computed to identify poorly scoring questions, indicating potential problems with the app.

## Phase 1: Icon Selection

A word-based menu-driven interview protocol designed to measure UEWCBs and tobacco and alcohol use in college-enrolled young adult MA women [12] was used as the starting point for the app development. The first step in the process was to replace word-based representations for the target UEWCBs, tobacco use, and alcohol use behaviors with icons or pictures that were culturally appropriate and easy for our participants to understand. In addition, we sought to add to the protocol measures of the emotional and social context in which the target behaviors occurred. To achieve these aims, a collection of icons and pictures to represent the target behaviors, moods, and contexts were gathered through Web searches completed by the research team. Icons were selected to test a variety of dimensions, such as cartoons versus realistic drawings versus photos, and items shown with and without women. The purpose of phase 1 was to validate the cultural appropriateness and understandability of the selected icons and pictures with the target population.

### Sample

Participants for this phase were 8 women recruited from farming communities in Western New York. All were first generation (eg, born in Mexico) and spoke Spanish as their primary language. The mean age of the sample was 28.3 years (range 21-32 years). Three participants had less than a high school education, 4 completed high school, and 1 completed some college. The mean SAHL score for the sample was 16.1 (range 13-18) with 3 women scoring in the range of low literacy (ie, ≤ 14).

### Phase 1–Specific Methods

Participants were asked to complete 3 card-sorting tasks to determine meaningfulness, cultural appropriateness, and preferences for the visual images. In task 1, participants were verbally provided definitions of all UEWCBs, tobacco use, and alcohol use behaviors (Table 1, left-hand column), and asked to sort icons into stacks of specific behaviors. Definitions were based on the *DSM-IV* and items from the Eating Disorder Examination [20] but were simplified to improve comprehensibility. Detailed properties of the behaviors were not included in the verbal definition, but plans were for the app to address them with follow-up screens. Once completed, we showed correct icons of each behavior together and asked participants to pick best, worst, and inappropriate icons for each behavior with 2-8 icons of each behavior shown.

In task 2, participants were asked to rate photos and drawings of emotions (happy, sad, angry, frustrated, worried, scared, lonely, and disgusted), locations (car, restaurant, field, store, bed, kitchen, living room, house, and church), activities (resting, working, socializing, exercising, eating, watching television, on the phone, driving, and on computer), and social context (alone, family, friends, coworkers, and crowd) depicted in another set of icons.

**Table 1:** Unhealthy eating and weight control behaviors, alcohol, and tobacco vocabulary and definitions used in the study.

Ten definitions before phase 2: focus groups	Eleven definitions after phase 2: focus groups
Exercise: workout, train, or exercise for the purpose of controlling your weight or shape or to burn calories.	Exercise: physical activities or exercise that you do to lose weight or avoid gaining weight.
Restricting: purposefully limiting the amount of food or calories that you ate or dieting to control your shape or weight. This includes the use of lower calorie meal replacement products such as Slim-Fast.	Changing what you eat or how you eat (diet): changing what or how much you ate to lose or control your weight or deal with your worries about being or getting fat. This would include cutting down how much you ate, cutting out certain foods, skipping meals, and not eating anything for a period of time.
Fasting: taking in no food for a period of several hours to control your shape and weight.	
Binge eating: you’ve eaten so much food in a short period of time that you would be embarrassed if others saw you, and during these times when you ate this way you feel you couldn’t stop eating or control what or how much you were eating.	Eat and eat: overeating, eating a large quantity of food that makes you feel guilty or embarrassed, accompanied by feelings of being out of control.
Diet pills and appetite suppressants: use of pills or other substances for the purposes of burning calories, altering your appetite or metabolism for the purpose of controlling your shape and weight.	Pills: pills that you take because of your worries about your weight and shape. Pills that cause you to get rid of food, reduce your appetite, lose weight, or avoid gaining weight.
Laxatives: taking pills or other types of substances to make your bowels move, to get rid of food or calories to control your shape or weight. Examples: pills, liquids, suppositories, enemas. Common brands may include but are not limited to Ex-Lax, Docusate Sodium, Metamucil, Milk of Magnesia, Citrucel, Fleet.	Powders: powders used to get rid of food, reduce your appetite, or cause you to lose weight or avoid gaining weight.
Diuretics: water pills or other substances for the purpose of reducing the amount of water in your body to control your weight or shape. This includes herbal pills and teas taken to reduce water weight.	Tea: tea that you drink to get rid of food, reduce your appetite, or cause you to lose weight.
	Drinks: drinks that are used to help you lose weight, sometimes they are taken instead of eating a meal; other drinks may be used to reduce your appetite or help you get rid of food.
	Drops: liquid drops that are put into food or water to help you get rid of food, reduce your appetite, or cause you to lose weight.
Vomiting: throw up to control your shape or weight or to get rid of food.	Vomiting: making yourself throw up to get rid of food or avoid gaining weight.
Smoking: use of cigarettes.	Smoking: use of cigarettes or tobacco products.
Alcohol: use of any alcoholic beverages.	Alcohol: use of any alcoholic beverages.

Task 3 involved two behaviors of interest that require the recording of time (binge eating and exercise). We showed participants two, 6-icon sets depicting various times. Participants were asked to select an icon from each set to report 50 minutes of exercise.

After the 3 card-based tasks were completed, participants were asked about the desirability of audio help within the app.

### Results

Performance on task 1 and translated audiotapes of questions during the task revealed that participants (1) did not understand the definitions of the UEWCBs and (2) often did not understand the link between the definitions provided and the icons. In particular, all of the participants had difficulty distinguishing between fasting and restricting images, with the audiotapes revealing they considered fasting to be a (extreme) form of restricting. Likewise, 7 of the participants had difficulty distinguishing images of laxatives, diuretics, and appetite suppressants, with the audiotapes revealing that most simply thought of products that would help them lose weight. Furthermore, images were not always understood as representations of UEWCBs. For example, icons to represent diet pills were understood as a general picture of pills. Similarly, icons to represent self-induced vomiting to control weight were interpreted as “being sick.” Finally, the concept of binge eating was unfamiliar and no suitable translation was identified.

In task 2, participants accurately identified photos and drawings of all 8 emotions and preferred photos of humans depicting the emotions compared with line drawings. Participants had considerable difficulty correctly identifying pictures related to location, activity, and social context.

In task 3, five participants correctly responded to the time task using the clock image and 7 reported that the clock image was easier to read.

Finally, all 8 participants indicated they would like the ability to play the text shown on the pictures aloud. However, 4 of 8 indicated they would not want audio to be played when other people were around.

### Discussion

There were two primary findings that led us to change our research protocol. The first was related to our study methodology: the SAHL measure of health literacy was not adequate to capture the range of cognitive functions required by the study tasks. An adequate literacy score using SAHL simply means a participant can read health vocabulary out loud and recognize word meaning; however, SAHL does not test more complex comprehension and application of concepts. Five of our 8 participants had adequate health literacy according to their SAHL scores, yet all of our participants struggled with understanding UEWCB definitions and 3 had difficulty interpreting time values. An investigation of the literature led us to add NVS to our future protocols, as NVS can be used to measure more complex manipulation of information. Our hope was that NVS would be a more complete measure of health literacy skills required to use a mobile app.

The other important finding indicated that making the EMA app appropriate for a low-literacy and low-acculturated population was not as easy as translating the existing protocol from text to icons, because the language and mental models of the targeted behaviors differed between our population and a higher-literacy MA population. Indeed, behaviors identified and defined in the original protocol suitable for high-literacy MA women were unfamiliar to our target population. Furthermore, the study showed that icons and pictures related to social context were unclear and confusing. These results made us go back to our target group to determine how they think about weight control behaviors so we could accurately capture that in an EMA app.

## Phase 2: Understanding UEWCBs

To gain an understanding of the conceptualization and language of UEWCBs of our target population, 2 focus groups were added to the study protocol. The goals of these focus groups were to (1) describe the weight and shape concerns of Mexican women living in Western rural New York, (2) describe behaviors employed to lose or control weight, and (3) identify the terminology used for these behaviors.

Purposeful recruitment occurred through bilingual community leaders who are well known in the community for being advocates for the local Mexican population (eg, project community liaison and a nutritionist who works at a local health center that services the farm worker community). The community leaders identified potential participants based on eligibility criteria and their knowledge of community members. Potential participants were provided with information flyers, and within 24-48 hours, one of the bilingual study team members contacted them to answer any question and determine interest. Interested women were informed of the date, time, and location of one of the focus groups.

### Sample

Eligibility criteria included MA women between the ages of 18 and 45 years. Most participants immigrated or migrated to the United States, primarily spoke Spanish, and worked in agriculture directly on farms or factories related to the agriculture industry. All participants lived in rural communities located in Western and Central New York. Because assessing participant literacy levels would have necessitated individual administration of a written questionnaire that would have significantly increased the duration of the meeting time and burden of participation, we did not collect specific demographic information or conduct any literacy or cognitive tests. Each focus group was composed of 7-8 women.

### Methods

The project community liaison and a bilingual nutritionist who also lives and works in the participants’ community facilitated the focus groups. The principal investigator (KS) and project coordinator (NT) participated as observers. The focus groups were held in community locations and free childcare was offered. The informed consent process was completed in the group setting with verbal consent because of immigration issues. A semistructured interview format was followed for the group meetings with probes to encourage detailed responses. Both groups were conducted in Spanish and lasted approximately 90 minutes. Both were audiotaped.

### Results

The detailed findings of the focus groups are reported elsewhere (manuscript under review). Here, we summarize the findings most relevant to the design of the app. We found that protocol labels for behaviors were not meaningful to the target population. First, participants recognized behaviors involving a product in terms of product form rather than product outcome, for example, a “diet pill” as opposed to a “laxative.” Second, participants tended to describe the actual eating and weight control strategy rather than label it, for example, “eat and eat” versus “binge eat” and “eat smaller portions” versus “restrict.” The language of behaviors that emerged was as follows: change what you eat, eat and eat, powders, drops, teas, shakes, pills, vomit, and exercise. The right-hand side of Table 1 provides the UEWCB labels and definitions used for the remaining phases included in this project.

### Discussion

In order to make the EMA app usable by participants, the app must employ their language and concepts for behaviors. However, researchers need to be able to label each participant-recorded behavior with the original protocol terminology. For example, it is not enough for researchers to know that women take pills to lose or maintain their weight. Researchers are interested in the types of pills they take (eg, is it a diuretic, appetite suppressant, or laxative?). This has implications for public health interventions. Thus, the app has to record enough information about the product being consumed (or behavior being engaged in) to map it to the original terminology (Table 1, left column). This has implications for the app design, requiring users to record additional information after a high-level behavior is selected.

With this new conceptualization and vocabulary identified, we added another picture card portion to the originally planned navigation phase. This picture card portion was used to identify appropriate icons for the redefined and newly identified behaviors.

## Phase 3: Finalize Icons and Navigation

A new collection of images depicting UEWCBs and context was gathered by our research team and tested in phase 3. Participants also were shown an early version of the app on a mobile phone and asked to navigate the app to record specific UEWCBs. This early version used a linear navigation and the Android hardware Home button, as has been shown to be usable by other low-literacy populations [17].

### Sample

Participants were 11 women of Mexican origin (all first generation and Spanish speaking). The mean age was 29.1 years (range 20-36 years). The average years of education was 8.5 with 3 of the 11 women completing high school and 3 completing grammar school only. The mean SAHL score for the sample was 14.2 (range 8-17) with 4 women scoring in the range of low literacy on this measure. The NVS measure of health literacy was added to our protocol for this phase. The NVS scores ranged from 0 to 3 with a mean score of 1, suggesting that 7 participants had high likelihood of low literacy and 4 participants had possible low literacy.

### Methods

Part 1 of this phase was composed of tasks 1 and 2 described under phase 1. Revised definitions of UEWCBs based on focus group results and corresponding photos and drawings gathered from the Web were used as stimuli for task 1. Given the difficulty participants had understanding the images for context, new photos and drawing were tested. In part 2 of this phase, participants were asked to record behaviors on a preliminary version of the app installed on the mobile phone. Brief scenarios of UEWCBs were read aloud, and participants were asked to record the behavior described on the mobile phone app.

### Results

Results for the picture card portion of this third phase were much improved. Conceptions of UEWCBs and related photo images were understood with 7 of the concepts being unanimous and 5 of the concepts having a single woman disagree that the final selected photo was a good representation of the concept. However, meaning of photos and images to denote context continued to be problematic for the majority of participants. Furthermore, although participants could correctly identify all of the icons depicting emotions, in both this phase and the first phase, participants only ever indicated that they personally felt positive emotions.

Results from the navigation portion of our third phase indicate that the task of recording behaviors on the app was difficult for our population. We determined this was at least in part because of the way we structured the tasks. Initially, the scenarios were written in the first person asking the participant to imagine that they just engaged in a target UEWCBs. Participants had difficulty with the “imagining task” and could not transfer the example to the app. In some cases, participants would argue that they would never perform the behavior we just had them imagine (eg, “I would never vomit on purpose!”), whereas in other cases participants had difficulty shifting between internal and external states over time (eg, reporting on an emotion they felt immediately before a behavior, then subsequently reporting their physical location at the time of the behavior).

### Discussion

Although participants endorsed experiencing negative emotions in relation to their weight, shape, and eating behaviors in the focus groups, participants in phases 1 and 3 only indicated having positive emotions. It is unclear why this discrepancy exists, but is likely a result of the methodology. Phases 1 and 3 were conducted with a single participant and 2 researchers. The formal nature of the interviews and the perceived power imbalance between researchers and participant could have played a role. Similarly, participants could have wanted researchers to perceive them positively or simply could have been much more reserved in that setting. With the focus groups, participants interacted with each other much more than with the research team. It was a more relaxed atmosphere, with a lot of laughing and storytelling. Further research is needed to understand the exact ways in which the two methods affect how participants describe UEWCBs and its context, but other researchers should be aware of this difference when studying similar sensitive health behaviors.

On the basis of results of the navigation portion of this phase, a decision was made by the research team to narrow the scope of the app to focus only on recording UEWCBs, tobacco use, and alcohol use behaviors. We determined that recognition and recording of mood and context just before the behavior was complex and would render the app too demanding to be reliably used. We also decided that answering the questions around the navigability of the app would have to be done in situ, when participants were actually engaging in the behaviors of interest.

## Phase 4: In Situ Beta Test

We conducted a beta test with the target population to assess the usability of the app. We were most interested in determining if recording their UEWCBs with the mobile app was intuitive, if the reminders were appropriate, and if the participants were able to use the app throughout their normal day.

### Sample

Seven MA women were recruited. Five participants were born in Mexico but had been in the United States for an average of 14.6 years. The remaining 2 were born and raised in the United States. The average age was 28.8 years (SD 5.72 years). Four participants were employed at the time of the study; 1 packaged vegetables, 1 cultivated crops, 1 assisted in a day care center, and 1 worked as a certified nurse assistant. All participants except one had 2-3 children in the age range of 1 to 14 years. Four spoke English but the main language of communication with friends and family was Spanish. Two also spoke another Mexican dialect—Mestizo.

All Mexican-born participants had completed schooling in Mexico—2 had completed high school, the remaining 3 had only attended until middle school. All participants owned a mobile phone and all had used some mobile apps and mobile Internet. The most popular apps were “YouTube” and “Facebook.” One participant had used health and wellness mobile apps such as Weight Watchers and Healthy Women. On the basis of the NVS, 5 of our 7 participants had low literacy skills.

### Methods

Participants received training to perform basic phone operations such as turning it on and off, use the back key, and so on. They were then trained to use the mobile EMA app. More specifically, participants learned to enter various behaviors, listen to instructions, change their passwords, make audio recordings, and take photographs. A picture-based manual was used to reinforce the topics covered during training. On average, a training session lasted for 1.5 hours.

Participants were then given the phone (and the training manual) to record their behaviors. Since the beta study aimed at testing the usability of the app, we deployed it for only 5 days. To encourage women to use the study phone, all phones were network activated and participants could use the phone and texting capabilities for personal use.

Participants were instructed to record behaviors each time they occurred. In addition, they also had to respond to 3 app-generated signals each day. At the completion of the study, we met with participants to collect the phones and administer questionnaires on usability and usage of the app. Participants were paid US $3 per signal response (3 signals/day × 5 days with maximum total US $45). Signal response did not mean a participant had to record a behavior—they could indicate that no behavior had occurred. In addition, they received US $10 for completing the post-study usability questionnaire and a semistructured interview to elicit their feedback on the app (total compensation up to US $55).

### Results

The EMA data from the beta study indicate that participants were able to use the app to record their UEWCBs. The 7 participants recorded a total of 135 behaviors. All behaviors were recorded by participants from 2 to 27 times. The most complex behavior to record was binge eating, which was selected a total of 16 times. Of these, 11 of the entries had complete follow-up questions (ie, duration of behavior, feelings of embarrassment, loss of control, and context of behavior). Of the 5 instances where the follow-up questions were not completed, participants recorded other behaviors during those sessions, indicating that participants likely selected binge eating accidently, then returned to the main menu to select the correct behavior.

The usability questionnaire indicates that participants found the app easy to use, with none of the Likert items averaging below a 3 (on a 5-point scale). Table 2 lists the mean scores for the 7 items with a standard deviation more than 1, indicating there was disagreement among participants. Upon closer examination, the first 4 items had all been ranked neutral or above, except for a single participant. Transcriptions of the sessions with this participant indicated she had significant difficulty with the app. The last 3 items had more significant differences, with 2-3 participants rating the question negatively. This indicates that 3 participants selected the wrong icons when navigating the app, 2 did not want others to know they were using the app, and 3 did not often take pictures of products (eg, laxatives).

**Table 2:** Usability items with SD&gt;1 for beta study.

Likert usability questions with SD > 1	Average	SD
31. When I began using the cell phone, I had no idea what I was doing.^a^	4.00	1.15
7. Even after I used the application for several days, I needed a lot of help to use it.^a^	3.86	1.07
19. The phone application gave too many reminders.^a^	3.86	1.07
27. I remembered to take the phone with me wherever I went.	3.86	1.07
10. I picked the wrong picture many times.^a^	3.14	1.07
28. I didn’t want people to know I was using the application.^a^	3.14	1.21
16. I often took pictures of products.	3.00	1.67

^a^ Denoted reverse coding. Items have been reverse coded so a high score reflects high disagreement.

The yes or no questions in the usability questionnaire were all unanimously positive, except for 3, all relating to context of use. Five participants indicated they did not use the app in front of friends, 6 did not use it in front of strangers, and 4 did not use it at work.

Finally, transcripts of the exit interview indicate that participants were generally unable to register their behaviors right at the time they took place, electing to record in response to reminders or at fixed times during the day. They found the reminders useful and appreciated the 30-minute response window. Some participants also requested being able to select diet throughout the day, and not just at the end of day reminder.

### Discussion

On the basis of participant feedback, we modified the final app in two primary ways. First, we allowed participants to select time periods for reminders during the initial application setup with researchers. This would reduce the possibility that the reminders would occur during inconvenient times, such as when they were working. Second, the app initially asked about dieting behaviors only at the end of the day reminder, because prior research had simply examined if a person had dieted for a particular day or not [12]. However, participants wanted to record every time they consciously performed a dieting behavior (eg, skipping lunch was a separate action from reducing a portion during dinner), and not just wait until the end of the day. Thus, the final app design includes dieting behaviors in the main menu so women can record them at any time.

## Final Ecological Momentary Assessment App

**Figure 2:**
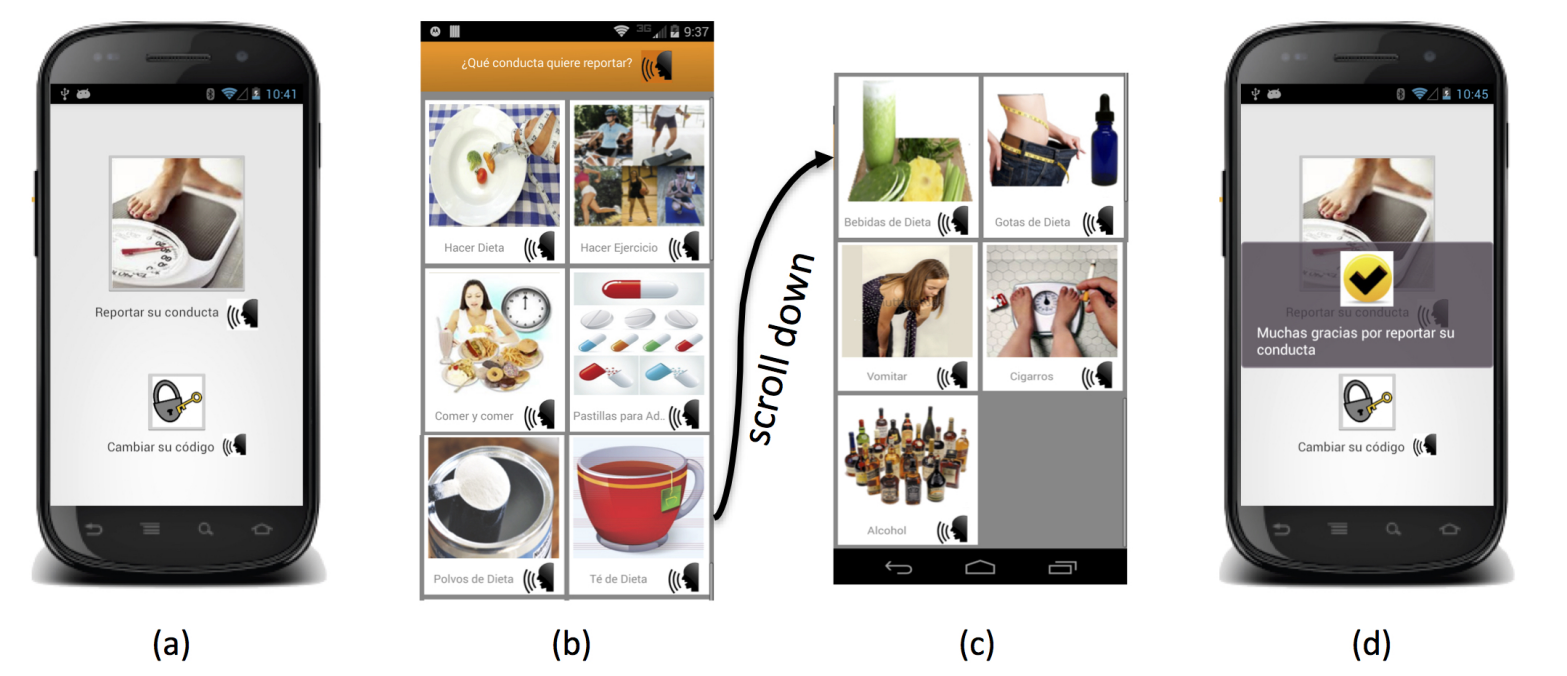
Screenshots of the interface: part (a) is the landing page and parts (b and c) are a selection of 11 behaviors to record (user must scroll to see items at the bottom). From top to bottom, left to right: changing what you eat or how you eat, exercise, eat and eat, pills, powders, teas, drinks, drops, vomiting, smoking to lose weight, and drinking. Part (d) shows acknowledgment that recording was successful.

**Figure 3:**
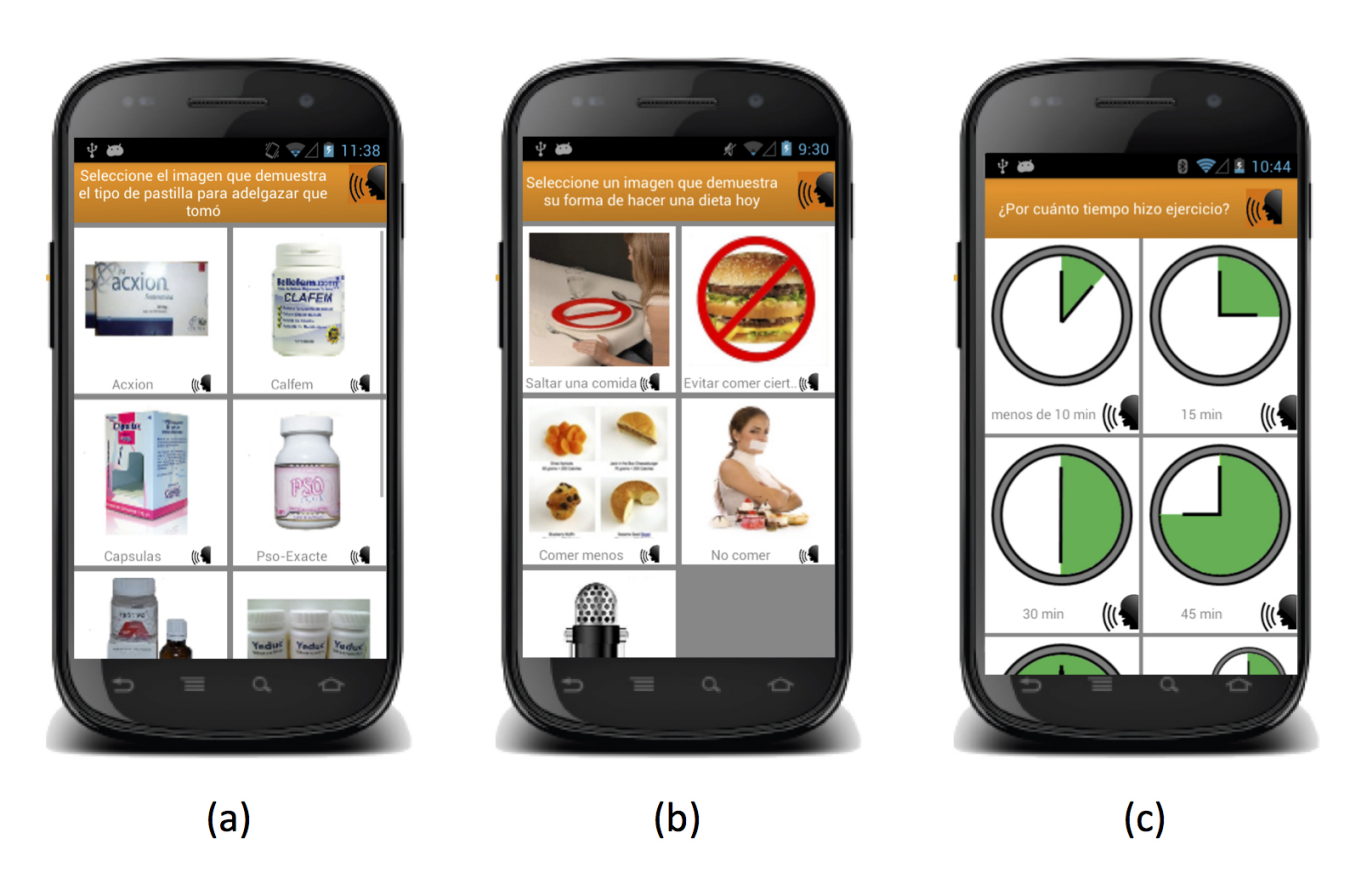
Screenshots of the interface: part (a) is a selection of pill products, (b) different types of dieting behaviors, and (c) time selection when recording exercise.

**Figure 4:**
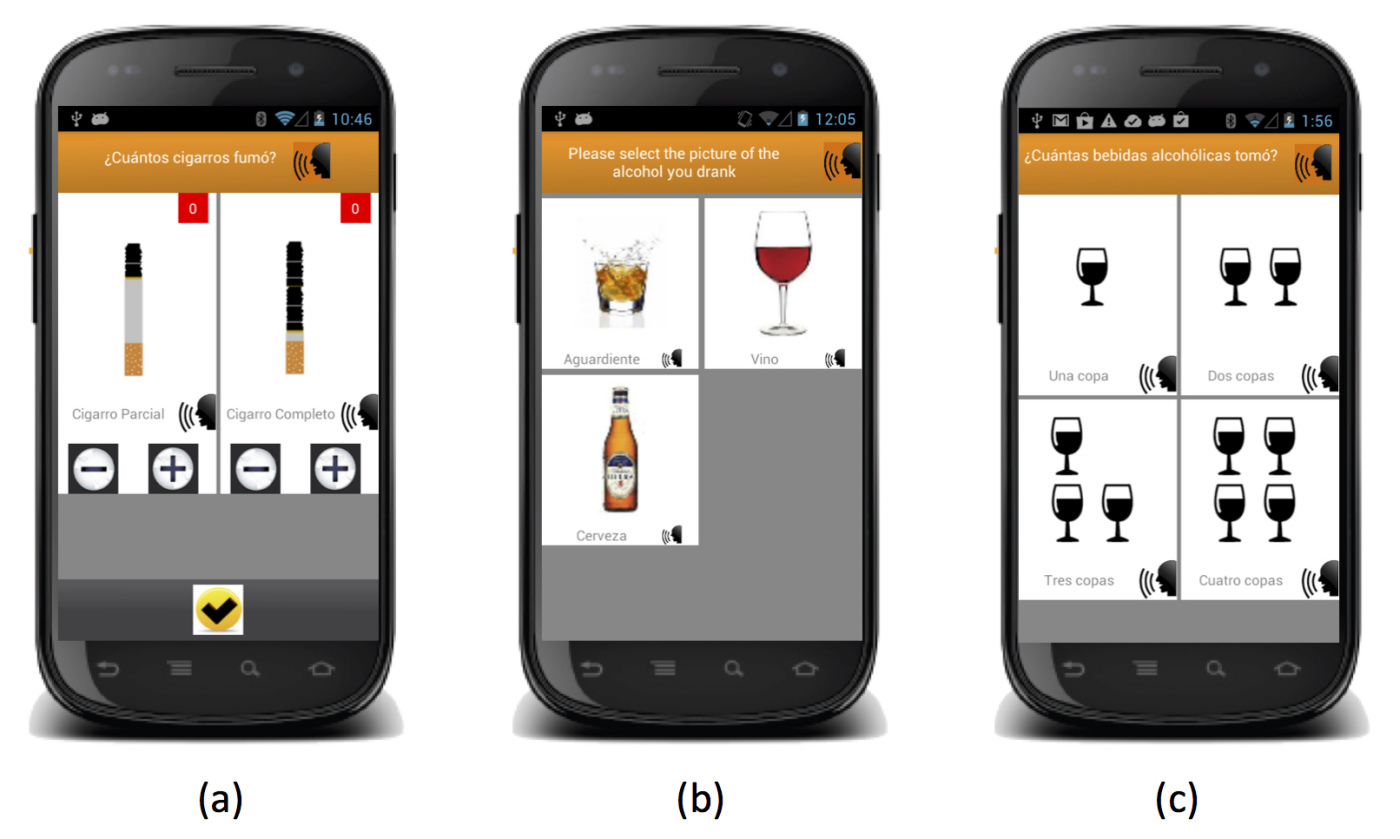
Screenshots of the interface: (a) recording the number of whole and partial cigarettes smoked; (b) different types of alcoholic drinks—mixed, wine, beer, and; (c) number of drinks when wine is being recorded.

**Figure 5:**
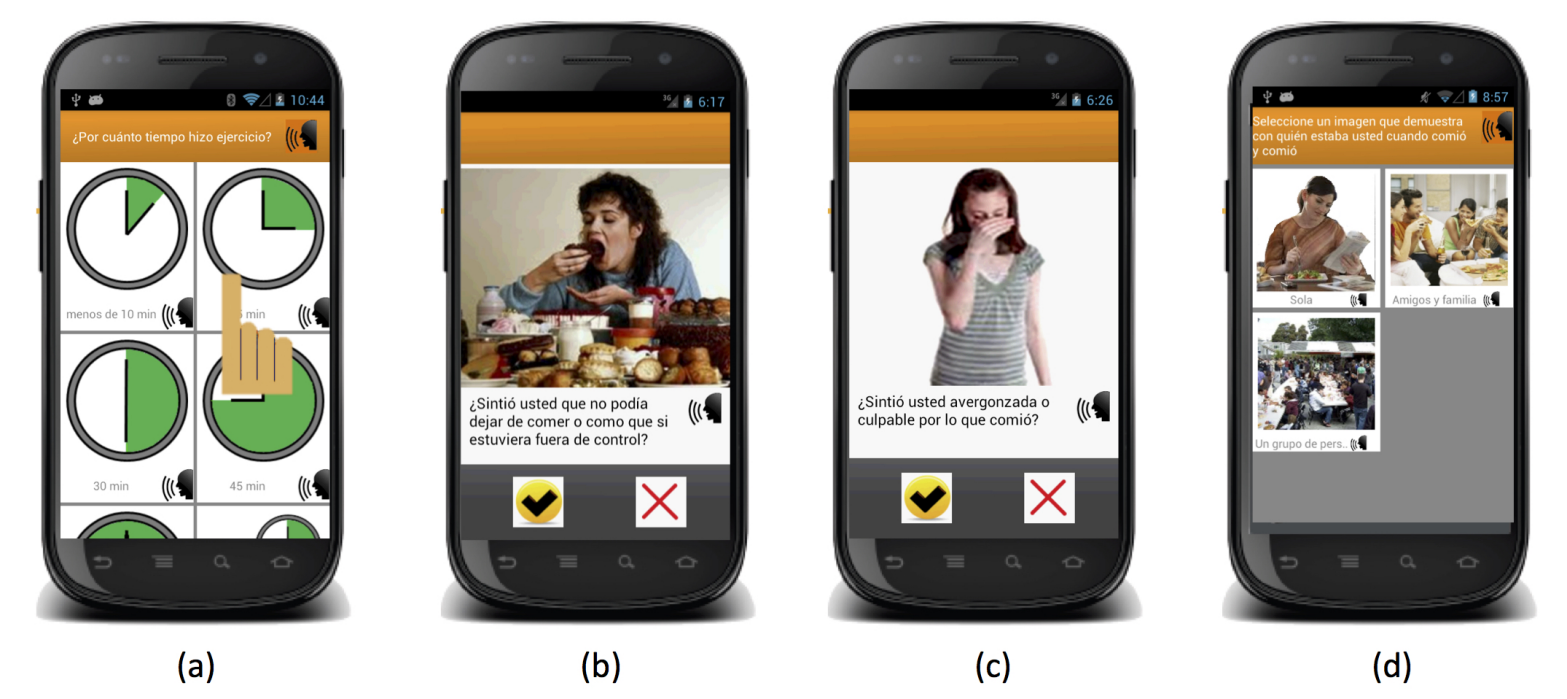
Screens to determine a binge eating episode, including (a) time interval in which eating occurred, (b) feeling a loss of control, (c) feeling embarrassed, and (d) who the person was with at the time of the episode.

The EMA mobile app was developed for Android mobile phones. Figures 2-5 show the interface. The landing page (Figure 2, part a) has an option to start a recording or to change their password to enter the app. On the recording page (Figure 2, parts b and c), the user can select from 11 options, which include the UEWCBs of interest, as well as alcohol and tobacco use, as described in the right-hand column of Table 1. Once a recording is complete, the user is returned to the landing page and an acknowledgment of the recording is briefly shown on the screen (Figure 2, part d).

Of all of the UEWCBs, only vomiting does not have any follow-up screens. The other behaviors prompt the user to select the specific product they consumed (eg, Figure 3, part a), record the kind of dieting behavior they engaged in (Figure 3, part b), the amount of time they engaged in physical activity (Figure 3, part c), the number of cigarettes or type and number of alcoholic drinks they consumed (Figure 4, parts a to c), or answer questions to determine if they engaged in an eating episode that met the criteria for a binge episode (Figure 5). The interface was primarily made up of large pictures with short Spanish labels. If a participant was unable to read the text, every label could be played out loud by pressing the speaker icon next to it.

## Discussion

Table 3 summarizes the major findings and their implications throughout all 4 phases. Here, we discuss the most significant findings that either changed the design of the EMA app or changed the way in which we performed the research with this population.

The iterative design process identified two significant findings that influenced the design of the app. First, low-literacy and low-acculturated MA women think differently about UEWCBs than researchers in three ways: (1) they used descriptions of the behavior instead of more formal terminology (eg, “eat and eat” instead of “binge eating”), (2) they did not differentiate between nuances of the same behavior (eg, restricting calories and fasting), and (3) they focus on form factor of a diet product instead of the underlying mechanism (eg, a pill, powder, tea, drink, or drops instead of appetite suppressant, laxative, or diuretic). The interface necessarily had to reflect the way the population thought about their behaviors in order to allow them to accurately record them. This required the team to make a mapping from the behavior or product selected by the participant (eg, a specific pill) to the UEWCBs of interest (eg, a laxative).

Second, the original protocol collected each behavior’s emotional, social, and locational context. However, from the initial user studies, we learned that women were lost when they could not find an icon matching their mental models or experiences of behaviors. We therefore simplified the protocol to collect type and description of behaviors only. Emotional and social contexts were required only for “eat and eat” (which corresponds to the formal definition of binge eating).

The design process also prompted us to refine our study protocols in two significant ways. First, we determined that a standard measure of health literacy (SAHL), which measures health vocabulary, was insufficient to differentiate between participants’ literacy and numeracy skills. The NVS measures the ability to translate health information to practice.

Second, performing in-lab user studies in which participants are asked to “imagine” a specific scenario before using the app was too confusing for our target population. Instead, studying the navigation of the app had to occur in situ.

Without an iterative (and flexible) user design process, the EMA app would likely have been too complex for our target population to use. The next step in our research is to use the EMA app with a much larger sample (n=60) of our target population for 3 weeks each, in order to document the prevalence of UEWCBs in this population. This will enable public health researchers to determine the types of interventions that are needed by low-literacy and low-acculturated MA women.

**Table 3:** Summary of major findings and their implications for each phase during the iterative, user-centered design process.

Phase	Finding	Implication/action item
**Phase 1: picture card study with 3 tasks**	Participants did not understand UEWCB^a^ definitions used by researchers.	Could not simply translate existing EMA^b^ protocol from text to pictures. Added focus groups to elicit their understanding of and language for UEWCBs.
	Participants accurately identified emotion icons but did not endorse negative emotions.	
	Participants had difficulty with location, activity, and social context icons.	Found more icons to test.
	Most participants could use the clock image for reporting length of time.	Include clock as part of EMA interface for recording binge eating and exercise.
	All participants wanted the ability to play audio to read text out loud.	Include option to play all text in audio.
	SAHL^c^ was not adequate for identifying cognitive abilities that affect participant ability to use a mobile app.	Added NVS^d^ to our protocol.
**Phase 2: two focus groups**	New language for some UEWCBs.	Used terminology familiar to participants in EMA app and training materials.
	Focus on form rather than function of UEWCB products.	Matched interface to mental model of participants. Had to expand application to gather enough information to map product form to function.
**Phase 3: picture card and navigation study**	Icons for new UEWCB terminology agreed upon.	Use appropriate icons in EMA app.
	Contextual icons still problematic.	Remove contextual questions *except* for binge eating.
	“Imagining tasks” were problematic.	Fully evaluate usability in situ.
	Participants only endorsed positive emotions, in contrast to focus groups.	Keep in mind that participants may be reluctant to record certain emotions.
**Phase 4: in situ beta study**	Participants could and did use the app to record a variety of UEWCBs in a variety of contexts.	
	Participants appreciated the reminders to record.	Retain reminders.
	Participants did not want to be disturbed during work.	Include customizable time periods for 3 reminders.
	Participants wanted to record every time they engaged in a dieting behavior, not just once a day.	Including dieting and restricting behaviors in main behavior page, not just end of day reminders.

^a^ UEWCB: unhealthy eating and weight control behavior.

^b^ EMA: ecological momentary assessment.

^c^ SAHL: Short Assessment of Health Literacy.

^d^ NVS: Newest Vital Sign.

### Limitations

Our design process was exclusively with MA women. Their UEWCB concerns and mental models are likely very different from other low-SES women, making many of the results very specific to this population. Because of privacy concerns and participant burden, demographic information was not collected for the focus groups in phase 2, although we are reasonably confident that the women do meet our inclusion criteria given the recruitment methods. Although the sample size was adequate for making design decisions, it was too small to perform statistical analysis for reporting, preventing us from correlating performance with literacy levels. Our subsequent field deployment will enable such analysis.

### Comparison With Prior Work

Most ESM studies assume adequate literacy and present the ESM questions in text form (eg, [2-4]). Ours is the first visual ESM implementation designed specifically for low literacy. Freedman and colleagues [21] performed an ESM study that was sensitive to the issue of low literacy, implementing their protocol through a phone-based menu system. They found that participants became frustrated with having to listen to the phone menu every time they called in and attempted to memorize the numbers associated with specific choices in an effort to skip the prompts. Because our system was visual, our participants could efficiently select the behavior they needed to record and did not experience the frustration of having to listen to a series of choices before finding the relevant one for a given recording session.

### Conclusions

There is a striking absence in the literature of using ESM apps to understand health behaviors of low-literacy populations. This paper provides evidence that an ESM app can be developed for those with low literacy. Emphasis must be placed on the interface mimicking how the population thinks about the behaviors, so they can easily record the behaviors of interest.

Although we were successful in designing an app for women to record a variety of UEWCBs, our attempts to record contextual information fell short. In particular, our participants could record the length of time for a particular behavior but had difficulty recording activity, location, and social context. Further research is required to determine the best way to obtain this type of context information from this population. A potential solution may be to automatically collect probable context with technology using Global Positioning System and state-of-the-art activity recognition algorithms. This would lessen the burden on participants and provide researchers much needed contextual information.

## Abbreviations

DSM-IV: Diagnostic and Statistical Manual of Mental Disorders, Fourth Edition
EMA: ecological momentary assessment
IRB: institutional review board
MA: Mexican American
NVS: Newest Vital Sign
SAHL: Short Assessment of Health Literacy
SES: socioeconomic status
UEWCB: unhealthy eating and weight control behavior
